# Supplementary material for: Full-length transcriptome profiling of Gentiana straminea Maxim. provides new insights into iridoid biosynthesis pathway
Source: PeerJ. 2025 Oct 23;13:e20136. doi: 10.7717/peerj.20136 (PMC12554311; doi:10.7717/peerj.20136)
Supplement: Supplemental Information 1 [file peerj-13-20136-s001.doc]

**Table S1** Sequence information of proteins analyzed in this study

| Organism | Sequence ID | Name in this study |
| --- | --- | --- |
| *Arabidopsis thaliana* | AtGPPS1 | NP 001031483.1 |
| *Pelargonium graveolens* | PgGPPS | ASQ40926.1 |
| *Gentiana straminea* | GsGPPS1 | CRA017932 |
| *Coffea arabica* | CaSPPS | XP_027089416.1 |
| *Coffea eugenioides* | CeSPPS | XP_027185499.1 |
| *Gymnema sylvestre* | GsyFPPS | UES73138.1 |
| *Sesamum indicum* | SiSPPS | XP_011096618.1 |
| *Nicotiana tabacum* | NtGGPPS3 | AHL84161.1 |
| *Catharanthus roseus* | CrGPPS1 | AGL91647.1 |
| *Catharanthus roseus* | CrGPPS2 | ACC77966.1 |
| *Catharanthus roseus* | CrGPPS3 | AHA82035.1 |
| *Coffea arabica* | CaGGPPS | XP_027075310.1 |
| *Coffea eugenioides* | CeGGPPS | XP_027178878.1 |
| *Gardenia jasminoides* | GjGGPPS | ARU08105.1 |
| *Catharanthus roseus* | CrGGPPS_LSU | AGL91645.1 |
| *Catharanthus roseus* | CrGGPPS2 | ACC77966.1 |
| *Swertia mussotii* | SmGPPS | ASK39399.1 |
| *Gentiana straminea* | GsGGPPS | CRA017932 |
| *Gentiana rigescens* | GrGPPS | AHK06853.1 |
| *Gentiana rigescens* | GrGPPS_LSU | ALS54746.1 |
| *Nicotiana tabacum* | NtGGPPS2 | NP 001312601.1 |
| *Mentha canadensis* | McGPPS_LSU | ABR15420.1 |
| *Gentiana lutea* | GlGGPPS | BAB82463.1 |
| *Catharanthus roseus* | CrGGPPS1 | AGL91648.1 |
| *Nicotiana tabacum* | NtGGPPS1 | ADD49734.1 |
| *Salvia miltiorrhiza* | SmilGGPPS | ACJ66778.1 |
| *Mentha canadensis* | McGPPS_SSU | ABR15421.1 |
| *Salvia miltiorrhiza* | SmilGGPPS SSUI | AEZ55678.1 |
| *Catharanthus roseus* | CrGPPS_SSUI | AGL91646 |
| *Nicotiana tabacum* | NtGGPPS_SSUI.1 | XP 016465609.1 |
| *Nicotiana tabacum* | NtGGPPS_SSUI.2 | XP 016455366.1 |
| *Pelargonium graveolens* | PgGPPS_SSUI | ASQ40929.1 |
| *Salvia miltiorrhiza* | SmilGGPPS SSUII | AEZ55679.1 |
| *Pelargonium graveolens* | PgGPPS_SSUII | ASQ40930.1 |
| *Corylus avellana* | CaveGGPPS_SSU | XP_059454032.1 |
| *Actinidia eriantha* | AeGGPPS_SSU | XP_057493525.1 |
| *Nicotiana tabacum* | NtGGPPS_SSUII.1 | NP 001312125.1 |
| *Nicotiana tabacum* | NtGGPPS_SSUII.2 | XP 016445326.1 |
| *Phtheirospermum japonicum* | PjGGPPS_SSU | GFP83728.1 |
| *Sesamum indicum* | SiGGPPS_SSU | XP_011092951.1 |
| *Olea europaea subsp. europaea* | OeGGPPS_SSU | CAA2982044.1 |
| *Gentiana straminea* | GsGGPPS_SSU1 | CRA017932 |
