# Supplementary material for: Full-length transcriptome profiling of Gentiana straminea Maxim. provides new insights into iridoid biosynthesis pathway
Source: PeerJ. 2025 Oct 23;13:e20136. doi: 10.7717/peerj.20136 (PMC12554311; doi:10.7717/peerj.20136)
Supplement: Supplemental Information 2 [file peerj-13-20136-s002.doc]

Table S2 Primer for real-time quantitative PCR

| Gene name | Primer name | Primer sequence(5’-3’) | Isoforms ID |
| --- | --- | --- | --- |
| AACT | FAACT331 | 5'-GCTACCATAATTGCTGCTCA-3' | Isoform0027033 |
|  | RAACT331 | 5'-TTTCGGCACATATTTCAGCA-3' |  |
| MVD | FMVD752 | 5'-ACAAGTCCCCTCATACAACA-3' | Isoform0014975 |
|  | RMVD752 | 5'-ATTCCACTTCTCAACACAGC-3' |  |
| IDI | FIDI032 | 5'-AGCGTGTTCCTGTTTAACTC-3' | Isoform0032003 |
|  | RIDI032 | 5'-AGTTTCCTTTGAGCAGCATT-3' |  |
| DXS | FDXS502 | 5'-TGAGATTTGCAATGGACAGG-3' | Isoform0015050 |
|  | RDXS502 | 5'-CTCGACTCCAATACCGTTTC-3' |  |
| ISPH | FISPH761 | 5'-AGGCGACTATACTTCTATCAT-3' | Isoform0026276 |
|  | RISPH761 | 5'-GCATACTTGAACTTCTCCAA-3' |  |
| GCPE | FGCPE991 | 5'-ATTCCTCTTGTGGCTGAT-3' | Isoform0014599 |
|  | RGCPE991 | 5'-TCCTTCTGATATTCGTCTTCT-3' |  |
| GPPS | FGPPS561 | 5'-CCAATTACCAGACCACCTT-3' | Isoform0027756 |
|  | RGPPS561 | 5'-CCGCCAGAACAGATAACTTA-3' |  |
| GAPDH | FGAPDH1 | 5'-TGATCTTGCCCATTCTGACG-3' | Internal reference |
|  | RGAPDH1 | 5'-AAACCGCACAACACTCCTCTAC-3' |  |
