# Supplementary material for: Full-length transcriptome profiling of Gentiana straminea Maxim. provides new insights into iridoid biosynthesis pathway
Source: PeerJ. 2025 Oct 23;13:e20136. doi: 10.7717/peerj.20136 (PMC12554311; doi:10.7717/peerj.20136)
Supplement: Supplemental Information 3 [file peerj-13-20136-s003.doc]

**Table S3 Gene function annotation via KEGG metabolic pathway classification**

| KEGG pathway classify | KEGG pathway classify | Number of Gene |
| --- | --- | --- |
| Cellular Processes | Transport and catabolism | 949 |
| Environmental Information Processing | Signal transduction | 795 |
|  | Membrane transport | 103 |
| Genetic Information Processing | Folding, sorting and degradation | 1482 |
|  | Translation | 1251 |
|  | Transcription | 748 |
|  | Replication and repair | 365 |
| Metabolism | Global and overview maps | 8924 |
|  | Energy metabolism | 1093 |
|  | Carbohydrate metabolism | 2909 |
|  | Amino acid metabolism | 1375 |
|  | Lipid metabolism | 1087 |
|  | Metabolism of other amino acids | 580 |
|  | Biosynthesis of other secondary metabolites | 460 |
|  | Nucleotide metabolism | 249 |
|  | Metabolism of terpenoids and polyketides | 288 |
|  | Glycan biosynthesis and metabolism | 368 |
|  | Metabolism of cofactors and vitamins | 477 |
| Organismal Systems | Environmental adaptation | 530 |
