# Supplementary material for: Full-length transcriptome profiling of Gentiana straminea Maxim. provides new insights into iridoid biosynthesis pathway
Source: PeerJ. 2025 Oct 23;13:e20136. doi: 10.7717/peerj.20136 (PMC12554311; doi:10.7717/peerj.20136)
Supplement: Supplemental Information 4 [file peerj-13-20136-s004.doc]

**Table S4** The ten pathways with the highest number of annotated genes in the KEGG

| Number | Pathway | Pathway ID | Count | All genes with pathway annotation (%) |
| --- | --- | --- | --- | --- |
| 1 | Metabolic pathways | ko01100 | 4647 | 48.99 |
| 2 | Biosynthesis of secondary metabolites | ko01110 | 2494 | 26.29 |
| 3 | Carbon metabolism | ko01200 | 826 | 8.71 |
| 4 | Biosynthesis of amino acids | ko01230 | 635 | 6.69 |
| 5 | Protein processing in endoplasmic reticulum | ko04141 | 588 | 6.2 |
| 6 | Spliceosome | ko03040 | 562 | 5.93 |
| 7 | Oxidative phosphorylation | ko00190 | 496 | 5.23 |
| 8 | Plant-pathogen interaction | ko04626 | 450 | 4.74 |
| 9 | Starch and sucrose metabolism | ko00500 | 449 | 4.73 |
| 10 | Endocytosis | ko04144 | 442 | 4.66 |
