# Supplementary material for: Full-length transcriptome profiling of Gentiana straminea Maxim. provides new insights into iridoid biosynthesis pathway
Source: PeerJ. 2025 Oct 23;13:e20136. doi: 10.7717/peerj.20136 (PMC12554311; doi:10.7717/peerj.20136)
Supplement: Supplemental Information 5 [file peerj-13-20136-s005.doc]

**Table S5** The number of genes involved in secondary metabolism according to the KEGG pathway analysis

| Pathway | Count | Pathway ID |
| --- | --- | --- |
| Phenylpropanoid biosynthesis | 84 | ko00940 |
| Terpenoid backbone biosynthesis | 121 | ko00900 |
| Ubiquinone and other terpenoid-quinone biosynthesis | 71 | ko00130 |
| Steroid biosynthesis | 53 | ko00100 |
| Carotenoid biosynthesis | 52 | ko00906 |
| Flavonoid biosynthesis | 32 | ko00941 |
| Nicotinate and nicotinamide metabolism | 47 | ko00760 |
| Tropane, piperidine and pyridine alkaloid biosynthesis | 36 | ko00960 |
| Diterpenoid biosynthesis | 5 | ko00904 |
| Isoquinoline alkaloid biosynthesis | 35 | ko00950 |
| Zeatin biosynthesis | 15 | ko00908 |
| Sesquiterpenoid and triterpenoid biosynthesis | 35 | ko00909 |
| Stilbenoid, diarylheptanoid and gingerol biosynthesis | 28 | ko00945 |
| Brassinosteroid biosynthesis | 13 | ko00905 |
| Flavone and flavonol biosynthesis | 3 | ko00944 |
| Monoterpenoid biosynthesis | 27 | ko00902 |
| Limonene and pinene degradation | 19 | ko00903 |
| Caffeine metabolism | 8 | ko00232 |
| Indole alkaloid biosynthesis | 18 | ko00901 |
| Isoflavonoid biosynthesis | 6 | ko00943 |
