# Supplementary material for: Full-length transcriptome profiling of Gentiana straminea Maxim. provides new insights into iridoid biosynthesis pathway
Source: PeerJ. 2025 Oct 23;13:e20136. doi: 10.7717/peerj.20136 (PMC12554311; doi:10.7717/peerj.20136)
Supplement: Supplemental Information 6 — “ –” i ndicatesnoE . C . number or KEGG Orthology ( KO ) [file peerj-13-20136-s006.doc]

**Table S6** Isoforms involved in iridoid biosynthesis

| Pathway | Gene name | Gene | E.C. number | KO | No. of isoforms |
| --- | --- | --- | --- | --- | --- |
| MVA | Acetyl-CoA C-acetyltransferase | AACT | 2.3.1.9 | K00626 | 5 |
|  | Hydroxymethylglutaryl-CoA synthase | HMGCS | 2.3.3.10 | K001641 | 8 |
|  | Hydroxymethylglutaryl-CoA reductase (NADPH) | HMGCR | 1.1.1.34 | K00021 | 17 |
|  | Mevalonate kinase | MVK | 2.7.1.36 | K00869 | 2 |
|  | Phosphomevalonate kinase | PMK | 2.7.4.2 | K00938 | 4 |
|  | Diphosphomevalonate decarboxylase | MVD | 4.1.1.33 | K01597 | 2 |
| MEP | 1-Deoxy-D-xylulose-5-phosphate synthase | DXS | 2.2.1.7 | K01662 | 13 |
|  | 1-Deoxy-D-xylulose-5-phosphate reductoisomerase | DXR | 1.1.1.267 | K00099 | 8 |
|  | 2-C-methyl-D-erythritol 4-phosphate cytidylyltransferase | ISPD | 2.7.7.60 | K00991 | 0 |
|  | 4-Diphosphocytidyl-2-C-methyl-D-erythritol kinase | ISPE | 2.7.1.148 | K00919 | 1 |
|  | 2-C-methyl-D-erythritol 2,4-cyclodiphosphate synthase | ISPF | 4.6.1.12 | K01770 | 0 |
|  | (E)-4-Hydroxy-3-methylbut-2-enyl-diphosphate synthase | GCPE | 1.17.7.1 | K03526 | 19 |
|  | 4-Hydroxy-3-methylbut-2-en1yl diphosphate reductase | ISPH | 1.17.7.4 | K03527 | 4 |
|  | Isopentenyl-diphosphate delta-isomerase | IDI | 5.3.3.2 | K01823 | 2 |
| Iridoid | Geranyl diphosphate synthase | GPPS | 2.5.1.1 | K14066 | 2 |
|  | Geranylgeranyl diphosphate synthase | GGPPS | 2.5.1.29 | K13789 | 8 |
|  | Geranyl diphosphate diphosphatase | GES | 3.1.7.11 | K20979 | 0 |
|  | Geraniol 10-hydroxylase | G10H | 1.14.14.83 | K15099 | 0 |
|  | Cytochrome P450 reductase | POR | 1.6.2.4 | K00327 | 5 |
|  | 10-Hydroxygeraniol oxidoreductase | 10-HGO | 1.1.1.324 | K23232 | 8 |
|  | Iridoid synthase | ISY1 | 1.3.1.99 | K22419 | 5 |
|  | 7-Deoxyloganetic acid synthase | 7-DLS(CYP76A26) | 1.14.14.161 | K20618 | 2 |
|  | 7-Deoxyloganetic acid glucosyltransferase | 7-DLGT(UGT8) | 2.4.1.323 | K21373 | 0 |
|  | 7-Deoxyloganic acid hydroxylase | 7-DLH(CYP72A224) | -- | -- | 0 |
|  | Loganic acid O-methyltransferase | LAMT | 2.1.1.50 | -- | 0 |
|  | Secologanin synthase | SLS | 1.14.19.62 | K13400 | 0 |
|  | Strictosidine synthase | STR | 3.5.99.13 | K01757 | 2 |

“--” indicates no E.C. number or KEGG Orthology (KO)
