# Supplementary material for: Full-length transcriptome profiling of Gentiana straminea Maxim. provides new insights into iridoid biosynthesis pathway
Source: PeerJ. 2025 Oct 23;13:e20136. doi: 10.7717/peerj.20136 (PMC12554311; doi:10.7717/peerj.20136)
Supplement: Supplemental Information 7 — Note: in G. straminea , GsGGPPS SSU denotes the small subunits of geranylgeranyl diphosphate synthase ; GsGGPPS represents the geranylgeranyl diphosphate synthase; GsGPPS indicates geranyl pyrophosphate synthase . [file peerj-13-20136-s007.doc]

**Table S7** Protein sequence homology of GsG(G)PPS

| Gene name | Species | Gene ID | Gene Abbreviations | Identity values |
| --- | --- | --- | --- | --- |
| GsGGPPS SSU | *Phtheirospermum japonicum* | GFP83728.1 | PjGGPPS SSU | 81.74% |
|  | *Actinidia eriantha* | XP_057493525.1 | AeGGPPS SSU | 81.55% |
|  | *Corylus avellana* | XP_059454032.1 | CaGGPPS SSU | 81.49% |
|  | *Sesamum indicum* | XP_011092951.1 | SiGGPPS SSU | 81.61% |
| GsGGPPS | *Catharanthus roseus* | AEI53622.1 | CrGGPPS | 74.06% |
|  | *Gardenia jasminoides* | ARU08105.1 | GjGGPPS | 71.28% |
|  | *Coffea eugenioides* | XP_027178878.1 | CeGGPPS | 72.04% |
|  | *Coffea arabica* | XP_027075310.1 | CaGGPPS | 72.29% |
| GsGPPS | *Coffea eugenioides* | XP_027185499.1 | CeSPPS | 91.84% |
|  | *Coffea arabica* | XP_027089416.1 | CaSPPS | 91.76% |
|  | *Catharanthus roseus* | AGL91647.1 | CrGPPS1 | 92.00% |
|  | *Catharanthus roseus* | ACC77966.1 | CrGPPS2 | 91.84% |
|  | *Gymnema sylvestre* | UES73138.1 | GsyFPPS | 91.53% |
|  | *Sesamum indicum* | XP_011096618.1 | SiSPPS | 90.68% |
|  | *Nicotiana attenuata* | XP_019262263.1 | NaSPPS | 90.75% |

Note: in *G. straminea*, GsGGPPS SSU denotes the small subunits of geranylgeranyl diphosphate synthase; GsGGPPS represents the geranylgeranyl diphosphate synthase; GsGPPS indicates geranyl pyrophosphate synthase.
