# Supplementary figures and images for: Full-length transcriptome profiling of Gentiana straminea Maxim. provides new insights into iridoid biosynthesis pathway
Source: PeerJ. 2025 Oct 23;13:e20136. doi: 10.7717/peerj.20136 (PMC12554311; doi:10.7717/peerj.20136)

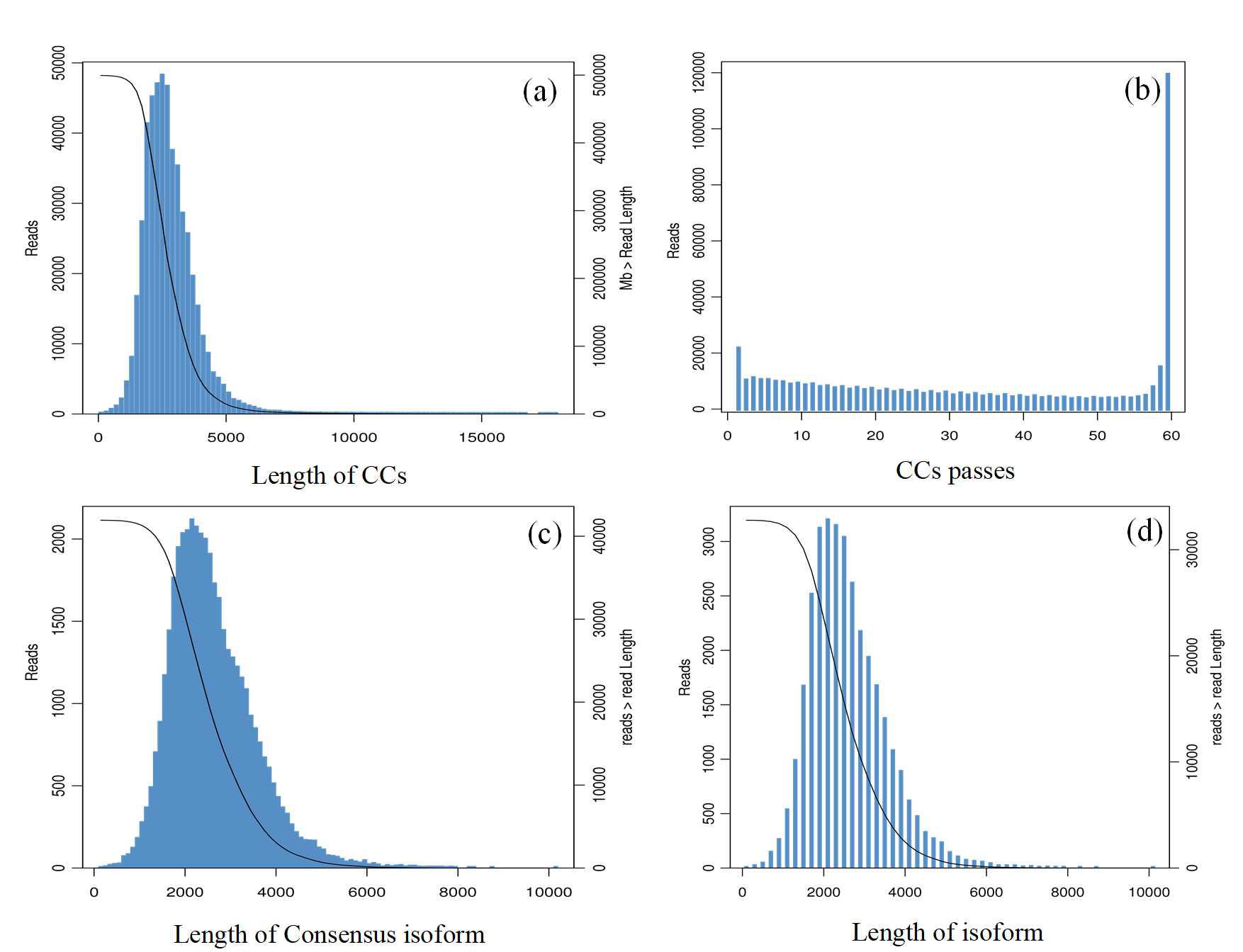

Supplement: Supplemental Information 8 — (a) CCS read length distribution; (b) CCS pass distribution; (c) Consensus isoform distribution; (d) Length distribution of transcript isoforms . [file peerj-13-20136-s008.jpg]

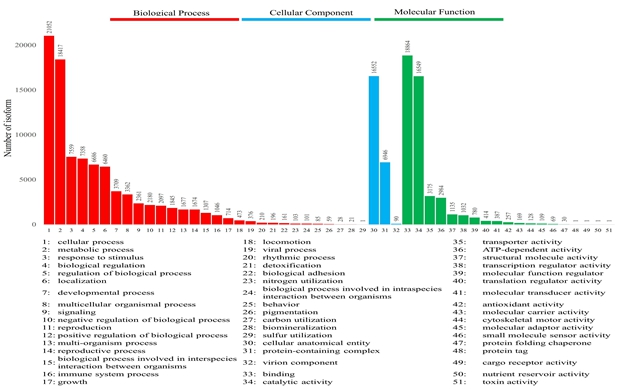

Supplement: Supplemental Information 9 [file peerj-13-20136-s009.jpg]

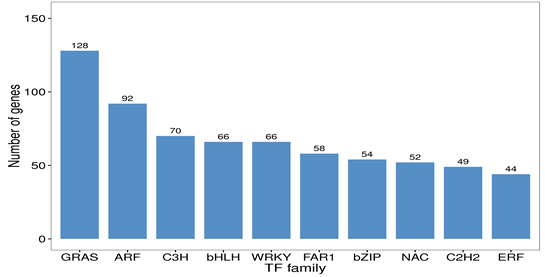

Supplement: Supplemental Information 10 [file peerj-13-20136-s010.png]

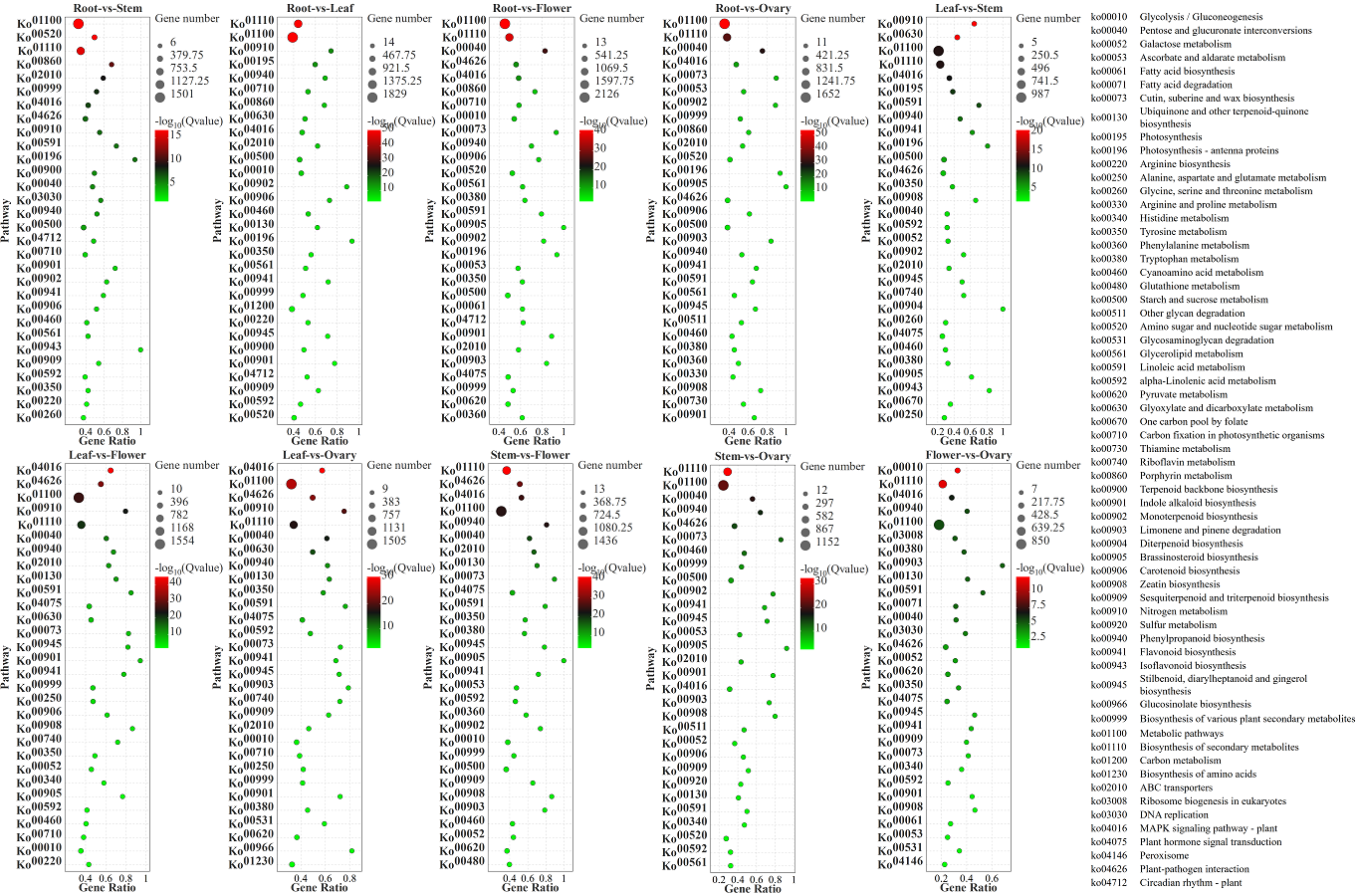

Supplement: Supplemental Information 11 [file peerj-13-20136-s011.png]

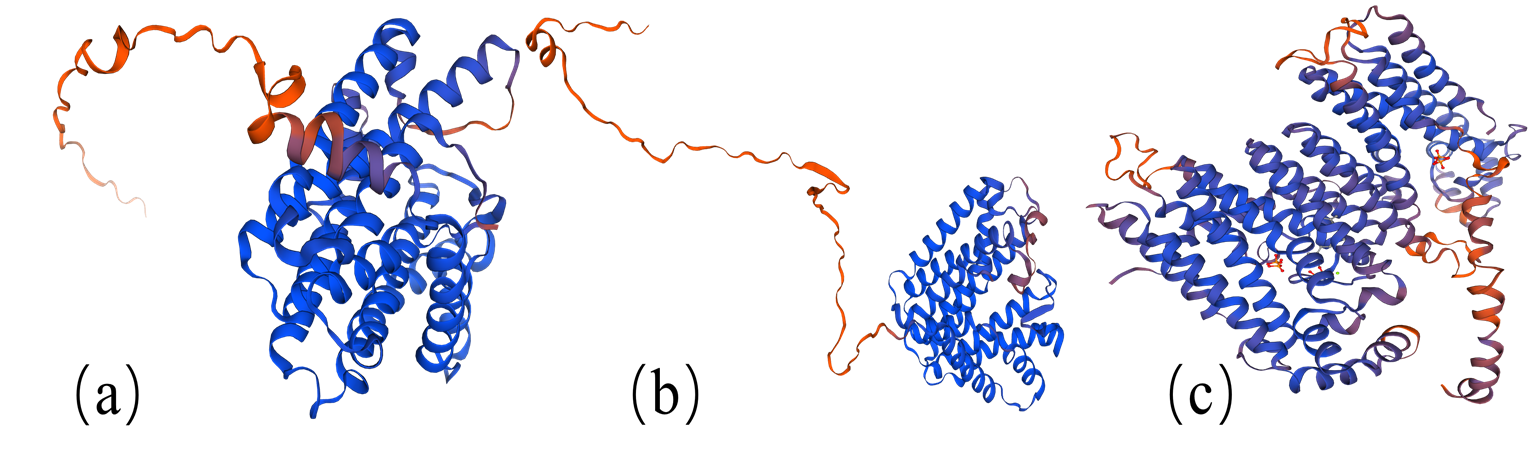

Supplement: Supplemental Information 13 — Structure prediction was performed by homologous modelling with the SWISS-MODEL sever, GsGGPPS SSU using structure of the geranylgeranyl pyrophosphate synthase small subunit protein of Mucuna pruriens (velvet bean) (A0A371F419) as the template. GsGGPPS using structure of the geranylgeranyl pyrophosphate synthase protein of Handroanthus impetiginosus GGPPS (A0A2G9GV50) as the template, GsGPPS using structure of the geranyl pyrophosphate synthase protein of Catharanthus roseus (B2MV87) as the template. (a) Predicted structure of GsGGPPS SSU shown; (b) Predicted structure of GsGGPPS shown; (c) Predicted structure of GsGPPS shown. [file peerj-13-20136-s013.png]
