# Supplementary material for: Full-length transcriptome profiling of Gentiana straminea Maxim. provides new insights into iridoid biosynthesis pathway
Source: PeerJ. 2025 Oct 23;13:e20136. doi: 10.7717/peerj.20136 (PMC12554311; doi:10.7717/peerj.20136)
Supplement: Supplemental Information 12 — (a) Sequence alignment of GsGGPPS(SSU) with GGPPS(SSU) from other plants; (b) Sequence alignment of GsGPPS with GPPS, SPPS from other plants; Identical residues are shaded in dark blue, highly similar residues are shaded in pink, and similar residues are shaded in light blue. Polyprenyl-synt domain was marked with purple border; DDXXXXD FARM and DDXXD (SARM) motif was marked with red border, CxxxC motif was marked with green border; Aspartate-rich region was marked with red Triangle; Chain length determination region was marked with green horizontal line; Active site lid residues was marked with blue horizontal line. Gene abbreviations, the species and gene ID information were shown in Table S1. [file peerj-13-20136-s012.pdf]

(a)

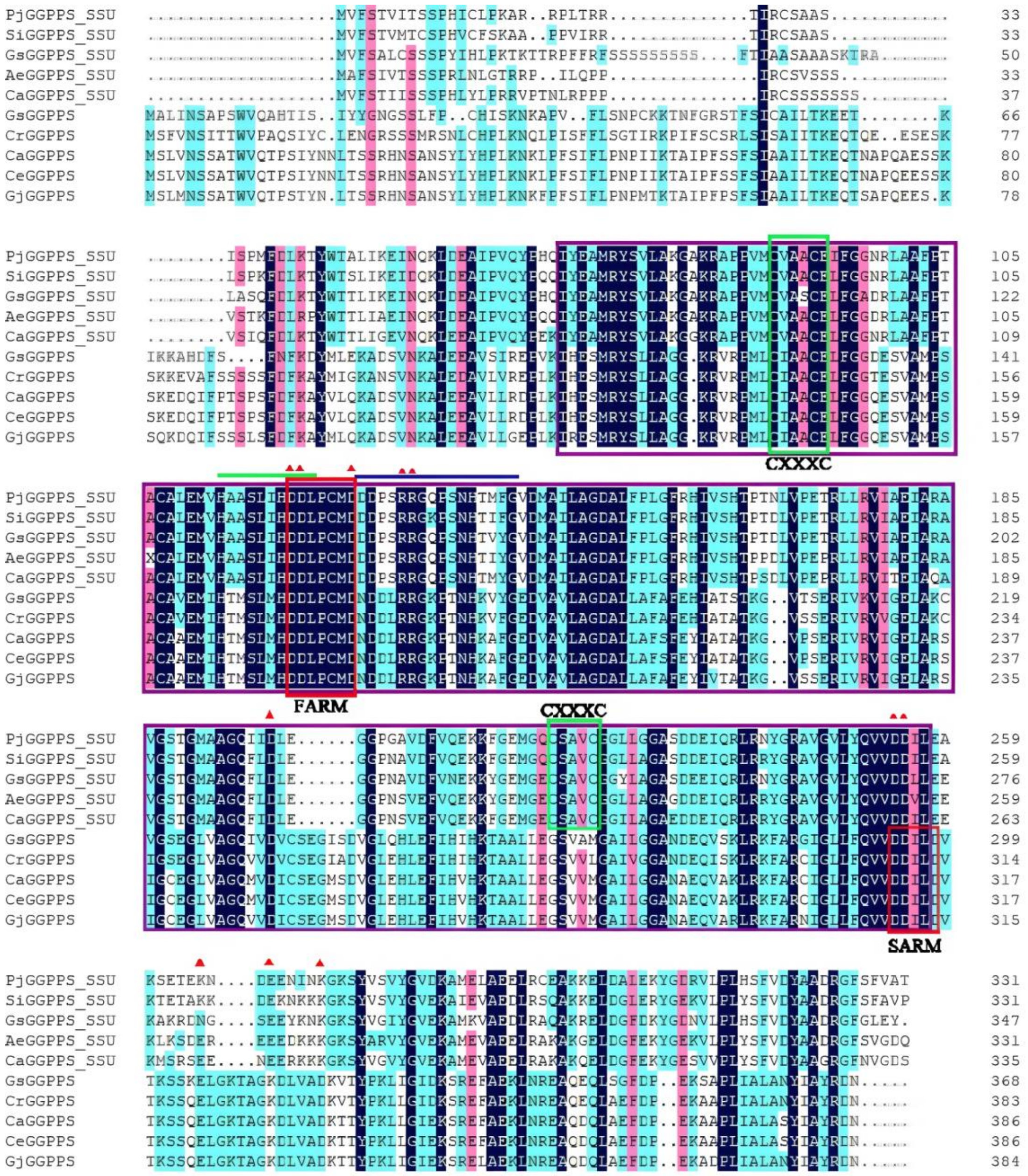
PjGGPPS_SSU
SiGGPPS_SSU
GsGGPPS_SSU
AeGGPPS_SSU
CaGGPPS_SSU
GsGGPPS
CrGGPPS
CaGGPPS
CeGGPPS
GjGGPPS
CXXXC
FARM
CXXXC
SARM

（b）

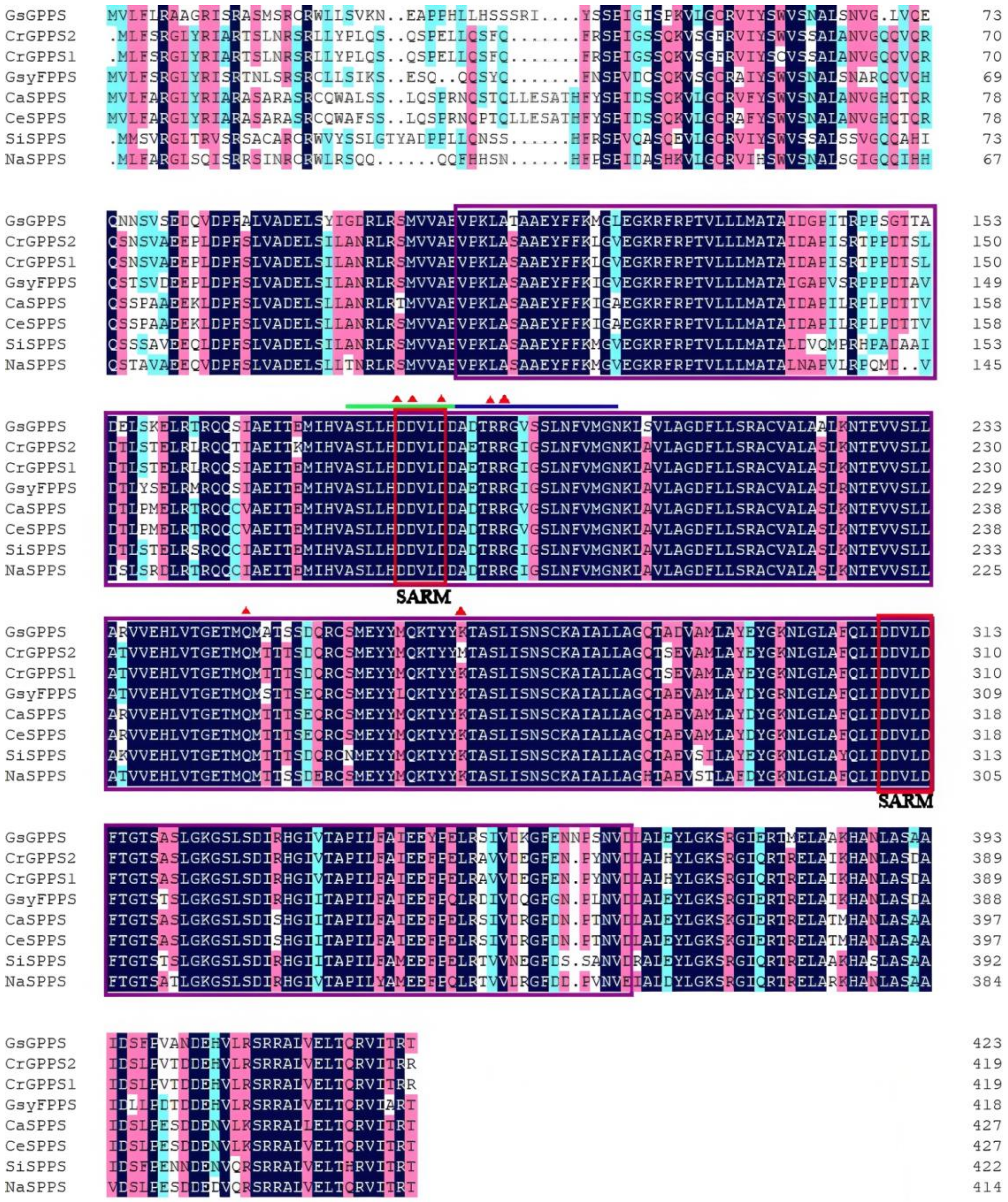
GsGPPS
CrGPPS2
CrGPPS1
GsyFPPS
CaSPPS
CeSPPS
SiSPPS
NaSPPS
SARM
SARM
