# Supplementary material for: Full-length transcriptome profiling of Gentiana straminea Maxim. provides new insights into iridoid biosynthesis pathway
Source: PeerJ. 2025 Oct 23;13:e20136. doi: 10.7717/peerj.20136 (PMC12554311; doi:10.7717/peerj.20136)
Supplement: Supplemental Information 14 [file peerj-13-20136-s014.docx]

**Processing procedure**

If frozen - how and how quickly? : By liquid nitrogen flash freezer

**Sample**

Sample storage conditions and duration：1 days at -80℃

**Nucleic acid extraction**

Nucleic acid quantification:

Instrument and method:We used NanoDrop 2000 to evaluate nucleic acid quantification

Purity (A260/A280): From 1.9 to 2.1

**Reverse transcription**

Complete reaction conditions

Amount of RNA and reaction volume

Priming oligonucleotide (if using GSP) and concentration

Reverse transcriptase and concentration

Step1. Prepare the following mixture in an RNase free centrifuge tube

| Oligo dT Primer(50 μM) | 1 μl |
| --- | --- |
| dNTP Mix (10mM each) | 1 μl |
| Total RNA | 1 μg |
| RNase-free ddH2O | Up to 10 μl |

Step2. 65℃ insulation for 5 minutes, then rapid cooling on ice

Step3. Prepare the following reverse transcription reaction solution in the above tube

| The above-mentioned denatured reaction solution ( from step 2) | 10 μl |
| --- | --- |
| 5 × PrimeScript II buffer | 4 μl |
| RNase Inhibitor (40U / μl) | 0.5 μl (20U) |
| PrimeScript II RTase (200 U / μl) | 1 μl (200U) |
| RNase-free ddH2O | Up to 20 μl |

Temperature and time

| 42°C | 45min |
| --- | --- |
| 95°C | 5min |

Storage conditions of cDNA: At -20℃

**qPCR TARGET INFORMATION**

Location of amplicon

Amplicon length：

AACT: 220bp; MVD: 216bp; IDI; 173bp; DXS; 215bp; ISPH: 192bp; GCPE: 152bp;

GPPS: 202bp.

**qPCR oligonucleotides**Manufacturer of oligonucleotides：Sangon Biotech(Shanghai)Co.,Ltd

**qPCR protocol**

Manufacturer of plates/tubes and catalog number：Sangon Biotech(Shanghai)Co.,Ltd F600384-0001

Location of amplicon ( highlighted in blue)

**AACT**

ATGGCTCCAGCAACAGCAGAGAGTATAAAGTCAAGAGATGTTTGCATTGTTGGTGTTGCC

CGCACACCGATGGGTGGTTTTCTTGGCACACTTTCTTCGTTATCATCAACTAAACTTGGA

TCTATAGCTATTCAAAATGCCCTTAAGAGGGCAAATGTAGACCCTTCTCTTGTACAAGAA

GTTTTCTTTGGAAATGTGCTCAGCGCGAACGTAGGGCAGGCTCCTGCTAGGCAAGCAGCA

ATAGGTGCAGGAATACCCAATACAGTGGTTTGTACTACAATCAACAAAGTATGTGCATCC

GGAATGAAAGCTACCATAATTGCTGCTCAAAGCATACAGTTGGGCATCAATGATGTAGTT

GTTGCTGGAGGCATGGAGAGCATGTCCAATGTCCCTAAGTATTTGGCAGATGCAAGGAAG

GGGTCTCGGCTTGGGCATGATACACTTGTTGATGGAATGTTGAAAGATGGTTTGTGGGAT

GTTTACAATGATTGCGGCATGGGAGTTTGTGCTGAAATATGTGCCGAAAGTCATGAAATT

TCAAGAGAACAACAGGATGATTATGCTGTTCAAAGCTTTGAGCGAGGCATTGCTGCTCAA

GAATGTGGTGCATTCTCATGGGAGATTGTTCCAGTTGAAGTGCCTGGTGGAAGGGGAAAA

CCTTCGATTGTTGTTGATAAAGATGAAGGTCTAGGGAAGCTTGATGCTGCAAAATTGAGG

AAGCTCCGTCCTAGTTTTAAGGAAACCGGAGGTACCGTTACTGCTGGCAACGCTTCCAGC

ATAAGTGATGGGGCAGCTGCTTTAGTTTTGGTCAGTGGTGAGAAGGCTCTTAGCCTTGGT

CTAACAGTAATTGCCAAGATCAGGGGGTACGCGGATGCTGCTCAGGCACCTGAACTATTT

ACCACTGCTCCTGCACTTGCAATTCCAAAAGCTATAGCAAATGCCGGCTTAGAGGCTTCT

CATATTGACTACTATGAGATCAACGAAGCCTTTGCGGTCGTGGCTTTAGCAAATCAGAAG

CTCCTTGGACTTAATCCTGAAAAGGTTAACGTACACGGTGGAGCTGTAGCATTGGGCCAT

CCTCTAGGATGCAGTGGAGCCCGTATCTTGGTCACACTTCTGGGGGTATTGAAACAAAAG

AACGGAAAGTATGGAGCAAGCGGTGTTTGTAATGGAGGAGGAGGTGCTTCAGCCCTTGTT

GTAGAGCTTCTGTAA

**MVD**

ATGGCTGTGGAATCTCAGAAGAAAGAGTCATGGGTTTTGACGGTTACAGCGCAAACGCCG

ACCAATATAGCGGTGATCAAGTACTGGGGGAAGAGGGACGAGTCCTTGATTCTTCCCATT

AACGACAGCATTAGTGTCACCCTCGATCCTTCCCACCTCTGCACAACGACCACTGTCGCC

GTCAGCCCTTCTTTCACTAATGATCGCATGTGGCTTAACGGCAAGGAGATCTCTCTATCA

GGAGGTAGGTATCAGAATTGCTTGAGGGAAATTCGGTCTCGGGCTTCTGATGTGGAGGAT

GCAGAAAAGGGAATCAAAATCACTAAGAAGGATTGGGAGAAGTTGCATCTGCACATTGCT

TCTTACAATAATTTCCCAACTGCAGCTGGTTTGGCTTCCTCTGCTGCTGGTTTTGCGTGC

CTTGTTTTTTCCCTAGCAAAGTTAATGAATGTGAAAGAAGACAATGGCCAACTTTCCGCT

ATAGCAAGGTAAGTCTTCTCTGTTATGGTGACTAGTTTTGATGTTTTGTCAGGATAATTG

TATGCGTTCTTCAGTTATACTCAAGGTCAGTAAGCTGCAGAAAAATATGTCGTGTATCCT

TTTTGAGAAGTGATCGGATATGTGTTGCTATTTTAACATGATGTATTGTATGTTAGATTA

GACTTTCATGTTTTTCGACTTCTCTTTCTCTACATTTATTTGGTTTTCCCTTTGTCTGTG

TTCATATGTCATCTGCTGAGAAACAACGTCTTAAAACTCCCTATTTTAATCAATTTTACC

TCACCTGATTAATATTATGATTCTTTGGAAAGCTCTGGTTTCATTCTCCTTCCCTTGCCT

TCTCTTATTGATTGATTTTTGACAAAAACTTTGAAGCCATAAAAGTAGTGCTTCTTGAAG

GTCCTCTCACCGCCTTTTTTCCTCTTTTTGTCAGAACCATTTATCTCTTATTGACCACAA

AACTACATTTGACTTTTGCATTTTGTATTTGGCCCTTCATATCAACCGTCCATCGCTGCT

GTTTGTATGGGGAAACCATTGAGATCACATGCCAAGTAACGGATCAGAAGCAGAACACTC

GATACGCAGAGAAAAGTGTGCAGCGGAGGAAAGACAAATTATTGGACCAGATTAAGTGGC

ATCATTGGTGGTTTTGGTTTTCATTAGCGTTCTTTAAACGTTGAAGTGTACTTCTTTTCG

AAGATTATTACAATGAACTATGGTTAATGAGCAGTGACAACTAGTTTTAGTATGAATATA

CATATAAATATGATTAGTTCATTAATTTTTATTTATTCAGGGGCATTAGTTCATTCACAA

AAGTAAATGAAGTTTATAAATCTCCTTTTCGATAGTGAATGGACATGAAAGGACACATCT

AGAAATTTCAGAAGGAAGCATATACTGAGAAGTAGATTGACAGACTTATTTATCCCGCTC

TGTAATTGCATTGTCAATTTCCATCAAGCCTGAGAAACTGAAAATTTTCTGCACGCTGTC

ATGTGATGTGGTTTCTTCCTAATTATTGGTGTATGTGTTTTATGTTTCTGAGACTTGAGA

CTAATGTAAGTTGCAGAATTGACTGAGATTTACTTTTGCAGGCAAGGTTCAGGAAGTGCT

TGCCGTAGCTTATATGGCGGATTTGTGAAGTGGATCATGGGAAAAGATGACAAAGGCAGT

GATAGCATAGCTGTTCAACTAGTGGATGAGAGTCACTGGGATGAGCTTGTTATTCTCATT

GCTGTGGTAAGTTCAAGGCAGAAGGAAACTAGTAGCACTAGTGGAATGCGGGAGACAGTT

GACACAAGTCCCCTCATACAACATAGAGCAGCTGAAGTAGTACCAAAACGCATACTTCAG

ATGGAAGAAGCCATAAAGAATCGGGATTTTCCAACATTTGCTCAACTGACTTGCTCAGAC

AGCAATCAGTTTCATGCTGTCTGCTTGGATACAAGCCCACCCATATTCTACATGAATGAC

ACATCCCATAGGATAATAAGCTGTGTTGAGAAGTGGAATCGTTCTGAAGGAACTCCACAG

GTGGCTTACACTTTTGATGCTGGGCCGAATGCAGTTTTAATTGCTCGTAATAGGAAGACT

GCTGCCTTACTGCTTCAAAGGCTGCTCTTTTATTTCCCTCCACAATCAGATGCAGATTTA

AACAGCTATATTGTCGGTGACAAATCAATACTTATAGATGCTGGCATTCAGGATATAAAA

GATATCGAAGCTTTGCCCCCACCCCCAGAAATAAAAGACAATATTCCTGCCCACAAGTTC

ACGGGCGATGTTAGCTATTTCATCTGCACAAAACCTGGTAAAGGTCCTATTTTGATTACA

GATGAAGATAAAGCTCTCCTCAGTCCTGAAACCGGTCTACCCAAGTGA

**IDI**

ATGGGCACTGTGGTTGAGGATTCCTCCATGGACGCCGTCCAGAAGCGCCTGATGTTTGAA

GACGAATGCATTCTGGTGGATGCGAATGATAACGTAGTTGGACATGACTCCAAGTACAATT

GTCACTTGATGGAAAAGATTGAATCTGAAAATTTGCTGCATAGGGCATTTAGCGTGTTCCTG

TTTAACTCGAAATACGAGTTACTTCTTCAGCAACGATCTGGTACGAAGGTGACGTTTCCTCT

CGTCTGGACAAATACTTGCTGTAGCCATCCTTTATACAGAGAATCTGAGCTGATTGAAGAG

AAATATCTTGGAGTGAGGAATGCTGCTCAAAGGAAACTCCTAGATGAACTCGGTATTCCTG

CTGAGGATGTCCCTGTTGATGAGTTCACTCCATTGGGTCGTATTCTATACAAAGCTCCATCT

GATGGGAAATGGGGAGAGCACGAACTTGATTACCTGCTCTTCATTGTGAAAGATGTTAAGG

TAAACCCAAATCCAGATGAAGTTGCTGATGCAAAATACGTGAATCGCGAGCAATTGAAGGA

TATACTGAGAAAGGCAGATGCAGGAGAAGAAGGTCTGAAACTTTCACCTTGGTTTCGACTA

GTCGTCGATAACTTCTTGTTCAAATGGTGGGACCATGTGGAGAAGGGTACCCTACAAGAA

GCTGCCGACATGAAAACTATCCATAAGTTGACTTGA

**DXS**

ATGGCGCTTTGTGGTGCATTTGCATTTCCGGTGAGCCTGAGCAAAACAGCTGTTTCAGAT

TCTGTAAAGCGTGATGCTTTGTACTCGTTCTGGCTCAATGGGACTGATCTGCAGTTTCAG

TGTCAATCCAGAAGCCTCCAGGACTCAAAGAAAGCCAATGGTGTAAGGGCATCACTTTCA

GAAAGGGAAGAATACTTTGCTCAAAGGCCACCAACTCCTCTATTGGACACAATTAACTAT

CCAATTCACATGAAAAACCTATCCAAAAAGGAATTGAAACAACTTTCGGATGAACTACGT

TCAGACATAATCTTTAACGTTTCAAAGACGGGAGGCCATCTTGGTTCAAGTCTAGGCGTT

GTTGAGCTCACTGTGGCTCTACATTATGTTTTTAACTGCCCACAAGATAAGTTCATTTGG

GATGTTGGCCATCAGTCTTATCCTCATAAGATTTTGACTGGGAGGAGAGATAAAATGCCT

ACAATGAGACAGACGAATGGGCTGTCTGGATTCACGAAGCGGTCGGAGAGCGAGTACGAT

TGCTTTGGGACCGGTCATAGTTCTACGAGCATTTCAGCTGGACTAGGAATGGCAGTTGGA

AGAGATCTAAAAGGGAGGAAAAACCATGTTGTAGCTATTATAGGTGATGGAGCCATGACA

GCAGGCCAAGCATACGAAGCCATGAATAATGCCGGTTACCTGGACTCCGACATGATCGTT

ATCCTAAACGACAACAAACAAGTCTCGTTACCAACAGCTACACTCGATGGACCTGCTCCT

CCGGTTGGAGCTCTAAGTAGCGCATTGAGTCGGTTGCAATCAAACAGACCACTTCGAGAA

CTCAGAGAAGTTGCTAAGGGTGTGACGAAACAGATTGGTGGACCGGTACACGAGCTTGCA

GCAAAAGTTGATGAATATGCTCGCGGTTTGATTAGTGGTTCTGGTTCGACACTGTTTGAA

GAGCTCGGGTTTTACTATATTGGCCCTGTAGATGGTCACAACATTGATGATCTTGTTGTC

ATTCTTCAAGAAGTCAAGAGTACGAAAACAACGGGTCCAGTACTAATCCATGTTGTTACT

GAGAAAGGCAGAGGCTATCCATATGCTGAAAAAGCCGCAGACAAGTACCATGGTGTGGTG

AAGTTTGATCCAGCAACGGGAAAGCAATTCAAATCGAGTCCAAGAACTCAGTCTTACACG

ACTTATTTTGCAGAGGCTTTGATTGCGGAAGCAGAGGTTGATAAAGACATTGTTGCTATT

CATGCTGCAATGGGCGGAGGCACGGGATTGAACCTTTTCCTTCGGCGTTTCCCTACACGG

TGTTTGGATGTCGGAATTGCAGAACAACATGCCGTAACTTTTGCTGCAGGATTGGCCTGT

GAAGGGCTCAAACCTTTTTGTGCTATCTATTCATCTTTCATGCAAAGGGCTTATGATCAG

GTAGTGCATGATGTGGATTTACAGAAACTGCCCGTGAGATTTGCAATGGACAGGGCAGGG

CTAGTTGGTGCAGACGGTCCCACGCATTGTGGTGCGTTTGATGTTGCGTTTATGGCGTGC

TTACCGAACATGGTGGTGATGGCTCCTTCGGATGAAGCCGAGTTGTTTCATATGGTAGCA

ACCGCAGCTGCTATAGACGATAGGCCTAGTTGCTTTCGGTACCCTAGAGGAAACGGTATT

GGAGTCGAGCTGCCACCTGGCAACAAAGGGGTCCCACTTGAGGTTGGAAAGGGCAGGATA

CTGATTGAAGGAGAGAGAGTGGTTCTTTTAGGTTACGGTACAGCAGTTCAGAGCTGTTTA

GCCGCAGCTACATTACTAGAAACACGTGGTTTACGGGTAACGGTTGCAGATGCGCGGTTC

TGTAAGCCGTTGGATCGTGGTATGATACGTAGCTTGGCTAAATCGCACGAGATCTTCATC

ACTATTGAAGAGGGCTCGGTTGGGGGTTTCGGGTCTCACGTTGCTCAGTTCATGGCCTTA

GACGGTCTTCTCGATGGCAACTTAAAGTGGAGGCCAATTGTTCTTCCGGATAGATACATT

GATCACGGATCTCCGGCTGACCAGGTGGCAGAAGCAGGTTTAACGCCAAGTCACATTGCA

GCAACGGTTTTCAACATTCTTGGACAAACAAGAGAAGCCCTAGAAATCATGTCATGA

**ISPH**

ATGGCAATCTCTTTGCAATTCGCTCGTCTCTCAGCCACTCCCTCGGCGGACCTCTCGTTG

CCGGCGGAGTCGAGGATATTTCGCTTCAGGAAACCTTTCTCTGTTCGATGTTCTGCTGCT

GGAGAAGCTCCATCGTCGTCTGTGAGTGTTGACTCGGAGTTTGACACGAAGAAGTTCAGG

CATGACTTGACTAGAAGCGAGATGTACAACCGCAAAGGATTTGGTCATAAAGAGGAAACA

CTTGAACTGATGAATCAAGAGTATACAAGTGACATTATGAAGGTTTTGAAGGAAAATGGA

TACCAATACACTTGGGGAAATGTAACCGTCAAACTTGCTGAGGCATATGGGTTCTGCTGG

GGTGTTGAGCGCGCTGTTCAGATTGCTTATGAAGCCAGAAAACAATTTCCAACCGAGAGG

ATTTGGATCACTAACGAGATTATTCACAATCCAACTGTTAACCAGAGGCTGGAGGACATG

GAAGTGAAGGAAATTCCTAAGGAGGAAGGAAAGAAACAATTTGATGTCGTGGAGAAGGGT

GACGTTGTGGTCTTGCCTGCTTTCGGGGCTGGTCTAGATGAGATGTTAACTTTGAGTGAT

AAAAAGGTTCAGATAGTTGACACTACTTGCCCATGGGTGTCTAAGGTTTGGACTAGCGTT

GAGAAACATAAGAAAGGCGACTATACTTCTATCATCCATGGCAAATATAATCATGAGGAG

ACAGTTGCCACCTCATCTTTTGCAGGAAAATATATCGTTGTAAAAAACATGAAAGAGGCA

ACATATGTTTGTGACTATATTCTGGGAGGTCAACTAGATGGATCTAGCTCAACCAAAGAG

GCATTTTTGGAGAAGTTCAAGTATGCAGTATCTAAGGGTTTTGATCCAGACACTGACCTC

ATAAAAGTTGGTGTTGCAAATCAAACAACGATGTTGAAGGGCGAAACAGAGGAGATTGGT

AAACTAGCTGAACGGACCATGATGCAAAAGTATGGAGTAGAAAATATCAACGAACACTTC

ATGAGTTTCAACACAATATGTGATGCAACACAGGAGAGGCAAGATGCTATGTATAAGCTG

GTTGACGAACCGGTGGATCTTATGCTAGTAATCGGAGGTTGGAACTCAAGCAACACATCA

CATCTGCAGGAGATAGCTGAGGATCGTGGAATCCCATCATATTGGATTGATAGTGAGAAG

AGAATTGGCCCTGGAAATAAAATAAGCTACAAATTAATGCATGGCGAGTTGGTTGAGAAA

GAGAATTGGCTACCCAAAGGTCCTATCACCATAGGGGTGACTTCTGGTGCATCTACTCCT

GATAAGGCCGTGGAAGATGTCCTCAACAAGGTTTTTGACCTTAAACGCGAAGAAGCCTTG

CAACTGGCTTAA

**GCPE**

ATGGCAAGTGGAGCTGTCCCAGCTTCTTTTACAGGTCTAAAGACCAATGAAAATGGATTG

GGGTTTGCTAAAAGTATGAATTTTGTTAAAGTATCTGACAAACAAAGAGTTAAATTTGTC

CGAAATAAGGTTTCTGTGATCAGAAATTCTAATCCTGGCCGGGAAACAGTTGAACTTGAG

CCTGCATCAGAAGGGAGTCCTTTATTAGTACCAAGACAAAAGTACTGTGAATCCGTACAC

AAAACCATCCGAAGGAAAACCCGTACTGTCATGGTTGGAAATGTGGGCATTGGTAGTGAT

CATCCCATACGGATTCAAACAATGACTACGTCAGATACAAAGGATGTTGCTGGAACAGTA

GCAGAGGTAATGAGAATAGCTGACAAAGGAGCAGATCTTGTACGAATAACAGTACAAGGA

AAAAAGGAAGCAGATGCTTGTTACGAGATAAAAAATACTCTTGTTCAGAAGAACTACAAC

ATTCCTCTTGTGGCTGATATTCATTTTGCTCCTTTGGTTGCTCTGCGTGTTGCTGAATGC

TTTGACAAAATCCGAGTCAACCCTGGAAACTTTGCGGACCGACGAGCTCAGTTTGAACAG

CTGGAATACACAGAAGACGAATATCAGAAGGAACTTGAGCATATCGAGCAGGTGTTTACA

CCTCTAGTTGAGAAATGCAAGCAATATGGGCGAGCTATACGAATTGGTACAAACCACGGA

AGCCTTTCAGACCGCATAATGAGCTATTATGGGGATTCCCCAAGGGGAATGGTGGAATCT

GCATTTGAGTTTGCTAGGATTTGCCGTAAATTGGATTACCACAATTTCGTGTTTTCAATG

AAAGCAAGCAACCCTGTTGTCATGGTTGAGGCCTACCGACTGCTCGTAGCTGAAATGTAT

GTTTTAGGATGGGATTATCCGTTACATCTTGGAGTTACAGAAGCTGGTGAGGGGGAGGAT

GGACGCATGAAATCTGCTATTGGTATCGGAACGCTTCTTCAGGATGGCTTGGGTGACACC

ATTAGAGTTTCGCTTACTGAACCACCGGAGGAGGAGATCGACCCCTGTAAAAGGTTGGCT

AACCTTGGTACAAGAGCTGCTGAGCTTCAGCAAGGAGTGGCGCCGTTTGAAGAGAAGCAC

AGACGTTATTTTGACTTCCAACGTAGGGCAGGTCAATTGCCGGTGCAATTGGAGGGGGAA

GAGGTGGACTATAGAGGTGTTCTACACCGTGATGGTTCTGTTCTCATGTCTGTTTCTCTG

GATCAGTTGAAGAATCCCGAAACTCTTTACAGATCATTAGCAGCAAAATTGGTGATCGGC

ATGCCATTTAAGGATCTTGCAACTGTGGACTCAATTTTGTTAAGAGAACTTCCTTCAGTT

GATGATAAAGACGCCAGACTAGCTCTAAAAAGGTTGATAGACATAAGCATGGGTATTATA

ACGCCTTTGTCCGAGCAATTAACAAAGCCTTTGCCGCACGCCATTGTTTTGGTAACTCTC

AAGGAATTATCGAGTGGAGCCCACAAGCTTCTACCTGAAGGAACACGGTTGGTTATATCT

GTACGTGGAGACGAACCAAAGGAAGAACTTGATATCCTGAAGAGCGTTCAAGCTACTATG

ATTCTTCATGATCTACCGTATACAGAAGTTAAAACTGGCAGGGTTCATGCAGCTAGGAGG

CTTTTTGAGTATCTTTCAGAAAACGCCCTTGATTTCCCTGTGATACATCACTTGCAGTTT

CCTAAAGGAATTCATAGGGATGACTTGGTCATTGGAGCCGGAAGCAATGCAGGAGCTCTT

TTGGTGGATGGTCTTGGAGATGGCCTCATGTTAGAAGCCCCGGATCAAGATTTCGAGTTT

CTGAGGAATACATCATTCAATTTACTGCAAGGTTGCAGAATGAGAAATACAAAAACTGAA

TACGTGTCGTGTCCATCCTGTGGACGGACTTTATTCGATCTGCAAGAGATAAGCGCAGAA

ATAAGAGAAAAGACATCCCACTTGCCTGGTGTTTCAATTGCAATTATGGGCTGCATTGTG

AATGGACCTGGTGAGATGGCTGATGCCGATTTTGGCTATGTTGGCGGTGCTCCTGGGAAA

ATCGACCTTTACGTAGGAAAGACGGTGGTCAAAAGAGGGATCGACATGTCACAAGCAACA

GATGCACTAATCCAGCTAATTAAGGATAATGGCCGCTGGGTTGAGCCTCCAACTGAAGAGTAA

**GPPS**

ATGGTTTTATTCTTGAGGGCAGCCGGTAGAATATCTCGGGCGAGTATGAGTCGCTGTAGA

TGGCTTTTATCGGTTAAAAACGAGGCGCCGCCACACCTTCTGCATTCTTCGAGCCGTATT

TATAGCTCTCCTATTGGTATTTCTCCAAAGGTTTTAGGTTGCAGAGTAATTTATTCTTGG

GTTTCAAATGCCTTAAGCAATGTTGGACTTGTACAGGAGCAGAACAATTCTGTTTCAGAG

GATCAAGTGGATCCATTTGCACTTGTTGCTGATGAACTGTCATATATTGGTGATAGATTG

AGGTCTATGGTAGTTGCAGAGGTGCCGAAGCTTGCCACTGCCGCTGAGTATTTTTTTAAG

ATGGGGCTCGAAGGGAAAAGGTTTCGGCCCACTGTTTTGTTATTGATGGCAACAGCTATT

GACGGGCCAATTACCAGACCACCTTCTGGAACAACCGCGGATGAGTTGTCCAAGGAGCTA

CGTACCAGGCAGCAGAGTATTGCTGAGATCACTGAGATGATCCATGTTGCTAGCCTGCTT

CATGATGATGTATTAGATGATGCCGACACTAGGCGAGGAGTCAGTTCATTGAATTTTGTA

ATGGGGAATAAGTTATCTGTTCTGGCGGGCGATTTCCTTCTATCTAGAGCATGTGTAGCC

CTTGCTGCTTTGAAAAATACGGAGGTTGTTTCCCTCTTGGCAAGAGTTGTAGAGCATCTT

GTCACTGGCGAAACAATGCAAATGGCAACATCATCTGATCAACGGTGTAGCATGGAGTAC

TATATGCAAAAGACGTACTACAAAACAGCTTCCTTAATTTCAAACAGTTGCAAGGCTATT

GCATTGCTAGCTGGACAAACTGCAGATGTTGCAATGTTAGCTTATGAATATGGAAAAAAT

CTGGGATTGGCATTTCAATTGATAGATGATGTCCTTGATTTCACTGGTACATCAGCTTCC

CTTGGCAAAGGTTCTTTATCCGACATTCGCCATGGAATTGTTACTGCTCCAATATTATTT

GCTATAGAAGAATACCCTGAACTACGTTCTATTGTTGATAAGGGATTTGAAAATAATCCT

TCAAATGTAGATCTTGCATTGGAATATCTGGGAAAAAGCCGGGGAATAGAAAGAACAATG

GAATTAGCAGCAAAACATGCTAATCTTGCTTCTGCTGCAATTGATTCTTTTCCTGTTGCA

AATGATGAACATGTTCTACGGTCAAGACGGGCTCTCGTAGAACTAACCCAAAGAGTCATT

ACCAGAACAAAATGA
